# Effect of maternal education on the literacy-numeracy development of children: A propensity-score matched analysis

**DOI:** 10.1371/journal.pone.0342297

**Published:** 2026-03-20

**Authors:** Md Akter Hosen, M. Iftakhar Alam, Mohaimen Mansur

**Affiliations:** Institute of Statistical Research and Training, University of Dhaka, Dhaka, Bangladesh; Rutgers The State University of New Jersey, UNITED STATES OF AMERICA

## Abstract

This study examined the causal relationship between maternal education and children’s literacy-numeracy development in Bangladesh using nationally representative data from the Bangladesh Multiple Indicator Cluster Survey. The analysis focused on 9,454 children aged 3–4 years. To strengthen causal inference, we applied propensity score matching, which reduces selection bias by balancing observed covariates between groups. From the analysis, we found that the average treatment effect of the treated was almost 18.4%, which means after matching, the probability of literacy-numeracy development in children was 18.4% higher among the mothers who had higher level of education compared to the same mothers had they not higher level of education. Subgroup analyses further revealed heterogeneity by place of residence: the effect of maternal education was stronger in urban areas (26.2%) than in rural areas (16.3%), suggesting that contextual factors may amplify or constrain the benefits of maternal education. These findings provide an evidence of the critical role of maternal education in early childhood development.

## 1. Introduction

Early childhood development (ECD) encompasses the physical, cognitive, social, and emotional growth and changes in children from birth through early adolescence. This phase is crucial for shaping a child’s future well-being, as foundational skills and behaviours are established during these formative years. The child’s rapidly developing brain is highly productive and dynamic from conception through infancy and childhood [1]. It represents a prime period for cultivating a strong interest in learning and developing the physical fitness required for later success and societal contribution [2]. Despite its significance, ECD still requires deeper understanding and often receives inadequate attention in many developing and low-income nations- such as Bangladesh, Nepal, and Nigeria- where investments in early childhood services remain limited [3,4]. In contrast, ECD has become a central component of health and education policies in many high-income countries, including Sweden, Australia, and the United Kingdom, which have implemented comprehensive early childhood care and education programs at the national level [5,6].

The UNICEF, in a recently published extensive report [7], highlights the need for protecting and supporting optimal nutrition, stimulation, learning and safe environment when defining ECD. It emphasises the importance of designing relevant policies and programmes, and good governance for achieving ECD targets, and outlines interconnections among these immediate, underlying and enabling determinants of ECD. In order to track ECD, UNICEF further calls for measuring children’s social, emotional, cognitive, language and motor development against internationally comparable data [8]. By the year 2030, countries that are members of the United Nations are mandated to guarantee that every child has access to high-quality early childhood development, as outlined in Target 4.2 of the Sustainable Development Goals (SDGs) [9]. However, the importance of ECD extends beyond this specific educational goal. It has relevance to various other SDG objectives associated with inequality, poverty and health, either directly or indirectly. Identifying factors that support or hinder development and implementing interventions based on these findings is essential for ensuring healthy and equitable childhood development within a nation.

Children facing impairments and cognitive challenges experience significant setbacks in early childhood development and learning [10]. McCoy et al. [11] highlighted that a substantial number of children worldwide fail to reach their developmental potential. The scale of the problem is more severe in the developing world. In low- and middle-income countries, approximately 43% of children under the age of five are at risk of not reaching their full developmental potential [12]. Frongillo et al. [13] conducted a study on 26 low- and middle-income countries and found family care practices and the nutritional status of children to be associated with ECD. Further studies on developing countries documented various other factors, e.g., iron and iodine deficiency, maternal depression, exposure to violence, poor home environment, inadequate cognitive stimulation as determinants of low child development [14,15]. In Bangladesh, about 64% of 3–4-year-old children were developmentally on track in 2012, and this figure increased to 74.5% in 2019 [16]. While this is a promising improvement, the extent of the problem is still concerning.

A child’s cognitive and noncognitive development is significantly influenced by the education level of parents, with a particular emphasis on the mother’s education [17]. In developing nations, social protection for children, encompassing aspects like nutrition, health and education, is generally more limited compared to industrialised nations. As a result, mothers often play a more substantial role in influencing children’s development. Bangladesh is among the ten nations where a significant number of underprivileged children face a high risk of delayed cognitive and social-emotional development [18]. There is evidence indicating that childhood abuse, a widespread but distressing issue in many underdeveloped nations, significantly hampers early childhood development [19]. Studies specific to Bangladesh consistently reported household wealth, parental education, regional variation, child punishment, learning environment and stimulation as key determinants of early childhood development in the country [16,20,21].

Literacy-numeracy is one of the four domains which constitutes the internationally comparable Early Childhood Development Index (ECDI) proposed by UNICEF for its Multiple Indicator Cluster Survey (MICS) [22]. Early childhood development in literacy and numeracy is vital for children, serving as the cornerstone for their holistic cognitive, social and academic development. The initial years of a child’s life- particularly from birth to age five- play a pivotal role in brain development, and engaging in activities related to literacy and numeracy fosters neural connections, contributing to the establishment of a robust cognitive base [8]. Moreover, acquiring proficiency in these skills equips children for formal education, providing a sturdy groundwork to help them successfully tackle academic challenges during their school years.

This paper explored the literacy-numeracy aspect of early childhood development, specifically examining how a mother’s education influences the literacy-numeracy development of 3–4-year-old children in Bangladesh. This study is conceptually grounded in both the human capital theory and ecological systems theory. Human capital theory posits that education enhances individuals’ cognitive and socio-emotional capabilities, enabling them to make informed decisions, manage resources effectively, and invest in the development of their children [23,24]. In this context, educated mothers are more likely to engage in developmentally supportive parenting behaviours, such as reading to their children, using rich vocabulary, and providing access to learning materials, thereby bringing up early literacy and numeracy skills. Complementing this, Bronfenbrenner’s ecological systems theory emphasises the importance of multiple, interacting environmental systems in shaping a child’s development [25]. Maternal education influences the child’s microsystem (e.g., quality of home learning environment and parent–child interaction), while also reflecting broader exosystemic and macrosystemic influences such as socioeconomic status and access to educational resources. By integrating these two theoretical perspectives, this study situates maternal education as both a personal and structural determinant of early childhood development, thus enriching the interpretation of the causal effects to be identified through the empirical analysis.

Several recent studies on Bangladesh and other developing and low-income countries found significant associations between maternal education and children’s literacy development (e.g., [16,20,26]). Alam et al. [16] found that maternal education was significantly associated only with the literacy-numeracy development of children. Specifically, after adjusting for other covariates, no significant associations were observed between maternal education and children’s physical, social-emotional, or learning development. This finding provides the rationale for our focused interest in the literacy-numeracy domain. However, we argue that these documented relationships are merely correlational as opposed to causal, because these studies predominantly model the literacy outcome using standard binary logistic regression which is not well-suited to estimate causal effects from observational studies, as educated and non-educated mothers are not balanced in terms of various socio-demographic characteristics [27]. By identifying these methodological limitations, this study employed cross-sectional data from the MICS 2019 and utilised propensity score matching to establish the causal impact of maternal education on childhood literacy and numeracy. Estimating such causal effects is important for justifying the need for implementing an intervention (e.g., promoting women education) for improving childhood literacy development.

While, to the best of our knowledge, propensity score matching has not previously been applied to examine the effect of maternal education on early childhood literacy-numeracy development in Bangladesh, the method is widely used in similar contexts in global research. Propensity score matching has been employed in various studies to estimate causal effects of parental education, health interventions, and early childhood outcomes from observational data where randomised trials are not feasible [28–30].

The paper’s organisation is as follows: Section 2, the data source, variables and methodology are described. Section 3 showcases the numerical findings, while Section 4 provides the corresponding discussion. Lastly, Section 5 contains the conclusions.

## 2. Methods

### 2.1. Ethics statement

This study utilised national-scale survey data from UNICEF, with all personally identifiable information of participants removed. The MICS conducted by the Bangladesh Bureau of Statistics under the guidance of UNICEF maintained a rigorous ethical protocol. Verbal consent was obtained from participants prior to the survey. All respondents were briefed on the voluntary nature of their involvement, as well as the assurance of confidentiality and anonymity of their information. Furthermore, participants were made aware of their right to decline to answer specific questions or to conclude the interview at any point. The dataset is available at the following link: https://doi.org/10.6084/m9.figshare.30076675.v1. Note that a substantial number of individuals in rural areas have low literacy levels. Asking for a written consent could make respondents uncomfortable or even discourage participation. MICS is conducted on a large national scale, requiring quick and efficient data collection. Written consent could slow down the process and add administrative burdens.

Since the analysis in this article did not involve direct studies involving human participants and was entirely based on freely available secondary data, this study did not require additional ethical approvals.

### 2.2. Data

The MICS initiative, launched by UNICEF in the 1990s, aims to assist countries in collecting internationally comparable data on various indicators related to the well-being of women and children. UNICEF created a module with an initial set of 48 items to collect information on six developmental domains of children. This module was first implemented in the third round of MICS (MICS3) for certain countries. Subsequently, the module underwent revisions and was streamlined to a 10-item format, focusing on four developmental domains: literacy-numeracy, physical, social-emotional and learning. The questions within the module were included in the questionnaire for children under five and directed to mothers (or caregivers) of 3 and 4-year-old children. The module was initially introduced in some countries during MICS4 but was adopted by Bangladesh in the fifth round of MICS in 2012. To ensure the reliability and validity of the module items, psychometric validation analyses were conducted at each stage of the development process, incorporating extensive literature reviews and pilot testing [22]. Utilising the information gathered through the module questions, an ECDI can be defined, representing the percentage of children who are developmentally on track in at least three of the four domains [22].

This paper utilised data obtained from the MICS, which was conducted by the Bangladesh Bureau of Statistics (BBS) in collaboration with UNICEF from January 2019 to June 2019 [31]. In the survey, mothers or caregivers of all children under the age of five in each selected household were given the questionnaire, and they provided responses to essential questions about their children. The data for 3- to 4-year-old children were extracted from the dataset on children under five, with a total sample size of 9,454 children aged from 3 to 4 years.

### 2.3. Outcome variable

This research focused exclusively on the literacy-numeracy domain of development. A child was considered to be on track in literacy-numeracy development if s/he could accomplish at least two out of the following three tasks: (a) identify/name at least ten letters of the alphabet, (b) read at least four simple, popular words (c) know the name and recognise the symbols of all numbers from 1 to 10. This literacy-numeracy ability was taken into account as a binary outcome variable.

### 2.4. Treatment and matching variables

In this study, we employed maternal education as the treatment variable, transforming it into a binary variable. Mothers with pre-primary, none, or primary education were categorised as ‘lower level of education’, while those with secondary or higher secondary education and above were classified as ‘higher level of education’. Note that, in the context of Bangladesh, primary education typically refers to grades 1–5, secondary education includes grades 6–10, and higher secondary education covers grades 11–12, based on the structure defined by the Ministry of Education, Bangladesh.

To assess the effect of maternal education on the literacy-numeracy development of children, it is crucial to account for confounders. Confounders are variables that influence both the mother’s education level and the child’s literacy-numeracy development. Mothers with different education levels may systematically differ in other characteristics, such as socioeconomic status, area, ethnicity, etc. which can also influence children’s literacy-numeracy development. For example, mothers with higher education are more likely to be from wealthier families or urban areas with access to better schooling facilities. If these factors are not accounted for, the observed effect of maternal education might actually reflect the influence of these confounders, not education itself. Therefore, in this paper, crucial socio-demographic confounding factors were taken into account for matching children of mothers with different education levels. These included community and household level variables: mother’s functional difficulties, area of residence, administrative division, household wealth index and ethnicity.

### 2.5. Propensity scores analysis

Randomised controlled trial is a gold standard for estimating causal effect of a treatment on an outcome. However, we often need to evaluate such cause-effect relationships from observational data where random assignment of treatment was not possible or not achieved or ensured. Propensity score analysis serves as a statistical method for examining observational data to evaluate the impact of a treatment while considering the factors influencing whether an individual receives the treatment. In observational studies with a limited number of events, propensity score analysis offers greater statistical power compared to traditional multivariable regression. This approach enables more effective control of confounding variables while mitigating concerns about overfitting or model convergence that are common in conventional regression analysis [27].

In this paper, we use a nearest neighbour matching algorithm for being the simplest algorithm. The process of pairing a given point with the ‘closest’ point in a matching issue is known as ‘nearest neighbour matching’. This is a ‘greedy’ algorithm, which cycles over each treated unit one at a time and chooses the available control unit that is closest to the treated unit. The definition of a distance measure is necessary for nearest-neighbour matching in order to determine which control unit is closest to each treatment unit. The default and most widely used measure is the propensity score difference, which is the difference between the propensity scores of each treated and control unit.

A propensity score is defined as the ‘conditional probability of assignment to a particular treatment given a vector of observed covariates’ [32], and is written as follows:


P(X)=Pr(A=1|X), 

where is *P*(*X*) is the conditional probability of having mother’s education, *A* = (0, 1) is exposure to mother’s education, and *X* is the vector of chosen socio-demographic characteristics associated with mother’s education. As discussed in the earlier section, these characteristics include mother’s functional difficulties, area of residence, administrative division, household wealth index and ethnicity.

Following a counterfactual paradigm, the estimation of the average treatment effect on the treated (ATT) can be expressed as follows:


$$ATT=E(Y_{1i}|A_{i}=1)-E(Y_{0i}|A_{i}=1),\;$$

where $Y_{1i}\;$ and $Y_{0i}\;$ are the two potential counterfactual outcomes of treatment and no treatment respectively. $E(Y_{1i}|A_{i}=1$ is the expected outcome of child literacy-numeracy of mothers with higher level of education, and $E(Y_{0i}|A_{i}=1$ is the expected outcome of child literacy-numeracy of mothers with higher level of education had none of the mothers received higher level of education (unobserved) [28,29,33]. The ATT can be conceptualised as the mean difference in a child’s literacy-numeracy development observed between mothers who have received education of higher level and those who have not received education of higher level.

## 3. Results

Table 1 presents the distribution of background characteristics of children, which includes literacy-numeracy status, mother’s education, mother’s functional difficulties, area of residence, division, wealth index, and ethnicity. It shows that 28.54% of children possessed literacy-numeracy skill, whereas 71.46% lacked this skill. Regarding maternal education, 61.93% of children had mothers with higher level of education, whereas 38.07% had mothers with lower level of education. Only 1.71% of mothers had functional difficulties, with the vast majority (98.29%) reported no such issues. In terms of residence, 81.43% of children lived in rural areas, compared to 18.57% in urban areas. The highest percentage of children were from Chattogram (20.99%), followed by Dhaka (19.12%), while the lowest was from Mymensingh (6.09%). The wealth index revealed that 25.49% of children were from the poorest households, while 16.16% were from the richest category. Additionally, the majority of children were Bengali (97.49%), with only 2.51% belonging to other ethnic groups. These distributions suggest potential disparities in educational outcomes linked to maternal education, area of residence, and socioeconomic status.

**Table 1:** Distribution of children by selected background characteristics.

Variables	Count	Percentage
Literacy-numeracy		
Yes	2,698	28.54
No	6,756	71.46
Mother’s education		
Higher level	5,855	61.93
Lower level	3,599	38.07
Mother has functional difficulties?		
Yes	158	1.71
No	9,095	98.29
Area of residence		
Rural	7,698	81.43
Urban	1,756	18.57
Division		
Dhaka	1,808	19.12
Barishal	827	8.75
Chattogram	1,984	20.99
Khulna	1,316	13.92
Mymensingh	574	6.07
Rajshahi	1,033	10.93
Rangpur	1,117	11.82
Sylhet	795	8.41
Wealth index		
Poorest	2,410	25.49
Poorer	2,006	21.22
Middle	1,770	18.72
Richer	1,744	18.45
Richest	1,524	16.12
Ethnicity		
Bengali	9,217	97.49
Other	237	2.51

Table 2 discloses that the ATT, representing the difference in outcomes between mothers who received higher level of education and the same mothers had they not received higher level of education, was approximately 18.4% (ATT = 0.184; 95% CI: 0.059–0.309). This implies that, after matching, the likelihood of literacy-numeracy development was 18.4% higher among children whose mothers received higher level of education compared to the same mothers had they not received higher level of education. In other words, having a mother with education increased the probability of early childhood development in literacy-numeracy by 18.4%.

**Table 2:** Average treatment effect on the treated of maternal education on early childhood development in literacy-numeracy.

Variable	Sample	Treated	Controls	Difference	S.E.	*T*-stat
Literacy-numeracy	Unmatched	1.348	1.183	0.165	0.009	17.24
	ATT	1.348	1.164	0.184	0.064	2.86

Table 3 presents model diagnostics. The likelihood ratio (LR) chi-square statistic is substantially large (1615.55) in the unmatched data, reinforcing that the covariates strongly predict treatment assignment. After matching, the LR chi-square statistic drops substantially to 158.31, reflecting an improvement in balance between the treatment and control groups. In both unmatched and matched samples, the *p*-value is 0.0000, confirming that the propensity score model is highly significant in predicting treatment assignment.

**Table 3:** Quality of matching and ATT of mother’s education on literacy-numeracy development of children.

	Model diagnostics			
	Pseudo $R^{2}\;$	LR chi2	*P* > chi2	ATT (95% CI)
Unmatched	0.1323	1615.55	0.0000	
Matched	0.1435	158.31	0.0000	0.184 (0.059-0.309)

**Fig 1:**
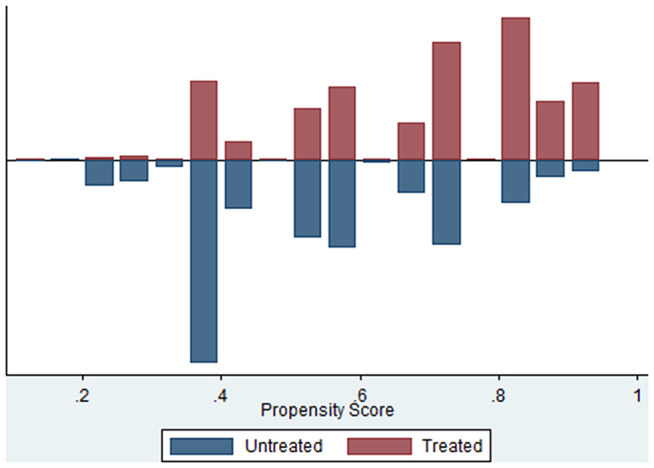
Propensity score distribution on treated and control group.

The distribution of propensity scores for educated and undereducated mothers is depicted in Fig 1. This illustration indicates a substantial overlap between the two groups, signifying that the common support condition is satisfactorily met.

**Fig 2:**
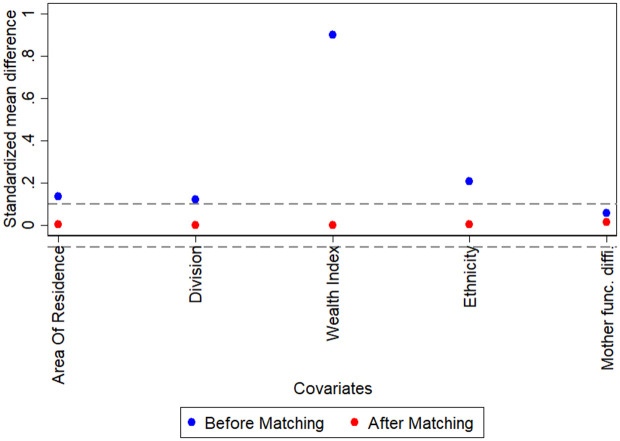
Standardised mean differences of covariates before and after matching.

Fig 2 presents the standardised mean differences (SMDs) of key covariates before and after propensity score matching. Prior to matching, several covariates-including wealth index, ethnicity, area of residence, and division-showed notable imbalance between the treatment and control groups, with SMDs exceeding the commonly accepted threshold of 0.1. After matching, however, the SMDs for all covariates were reduced substantially, falling well below the 0.1 threshold. This indicates that the matching procedure was successful in achieving covariate balance, thereby strengthening the credibility of the estimated treatment effects.

The Rosenbaum sensitivity analysis was conducted to evaluate the robustness of the estimated treatment effect to potential hidden bias [34]. The results show that across all tested values of Γ (ranging from 1.0 to 2.0), both the lower and upper bound *p*-values remain equal to 1.0. This indicates that even if unobserved confounding factors caused the odds of receiving treatment (mother’s education) to differ by as much as a factor of two between matched children, the positive effect on literacy-numeracy recognition would still remain statistically significant. In other words, the observed causal relationship is highly robust and unlikely to be explained away by hidden bias within this range of sensitivity.

The balance diagnostics indicate that nearest neighbour matching achieved satisfactory covariate balance. We acknowledge that alternative approaches, such as optimal or full matching, could also be employed. In our analyses, optimal matching yielded an ATT estimate of 0.183, while full matching produced an estimate of 0.179; the corresponding ATT from nearest neighbour matching was 0.184. Overall, the estimated treatment effects are highly comparable across the different matching approaches.

Subgroup analysis was conducted to explore potential heterogeneity in the effect of maternal education on children’s literacy-numeracy development. The effect of maternal education is unlikely to be uniform across populations, as contextual factors such as access to educational resources, socioeconomic opportunities, and cultural practices differ between rural and urban settings. Examining these subgroups separately allows us to show how the impact of maternal education varies across contexts, which in turn helps in designing policies for different populations rather than applying a uniform solution.

The subgroup analysis reveals that maternal education has a significant positive impact on children’s literacy-numeracy development, with stronger effects observed in urban areas compared to rural settings. In rural areas, the ATT indicates that children of educated mothers are 16.3% more likely to achieve literacy-numeracy milestones compared to their counterparts whose mothers have lower education; see Table 4.

**Table 4:** Average treatment effect on the treated of maternal education on early childhood development in literacy-numeracy for rural children.

Variable	Sample	Treated	Controls	Difference	S.E.	*T*-stat
Literacy-numeracy	Unmatched	1.330	1.181	0.150	0.010	14.44
	ATT	1.330	1.168	0.163	0.073	2.24

In urban areas, the corresponding effect is even larger, at 26.2%, suggesting that the benefits of maternal education may be amplified in environments with greater access to educational and social resources: see Table 5. These results highlight the critical role of maternal education in promoting early childhood literacy-numeracy outcomes across both rural and urban contexts, with particularly pronounced advantages in urban settings.

**Table 5:** Average treatment effect on the treated of maternal education on early childhood development in literacy-numeracy for urban children.

Variable	Sample	Treated	Controls	Difference	S.E.	*T*-stat
Literacy-numeracy	Unmatched	1.413	1.194	0.219	0.024	9.01
	ATT	1.413	1.151	0.262	0.121	2.18

## 4. Discussion

The literature review identified several studies exploring different aspects related to children’s literacy-numeracy development [35–37]. Research in the context of Bangladesh found positive association between children’s early literacy development and maternal education, among other factors (e.g., [16,38]). These studies, however, primarily employed observational methods and conventional regression techniques to identify factors related to such development. As far as we know, this paper represents the first instance in Bangladesh where propensity score matching is applied to estimate causal effect of maternal education on children’s literacy-numeracy development from nationally representative observational data and thus, makes an important contribution in investigating a crucial public health question.

The current study found a significant and positive effect of maternal education on literacy-numeracy development, following the matching of treated and untreated mothers based on all relevant observable characteristics. These findings support existing literature emphasising the influence of maternal education on children’s literacy-numeracy development. For instance, Alam et al. [16] found that maternal education was significantly associated with children’s literacy-numeracy development in Bangladesh using binary logistic regression, reporting adjusted odds ratios around 1.92 for children of mothers with secondary education or above. Similarly, Lim et al. [26], using MICS data from Ghana, reported that maternal education was a strong predictor of literacy-numeracy development, with adjusted odds ratio 1.25 for children of mothers with higher education. Since these studies did not use propensity score matching, a direct comparison between the ATT and odds ratio is not appropriate. Nevertheless, both measures consistently suggest that maternal education has a significant positive effect on children’s literacy-numeracy outcomes.

While the estimated 18.4 percentage point increase in literacy-numeracy development is statistically significant, it is also meaningful in practical terms. This improvement indicates that a substantially greater proportion of children with educated mothers are acquiring core pre-academic skills-such as letter recognition, basic word reading, and number identification-that are critical for early grade learning. Enhanced readiness in these areas supports better performance in primary school, facilitates smoother adaptation to formal educational settings, and may reduce future dropout risk [39,40].

Mothers who have attained higher level of education typically participate in more frequent and diverse language interactions with their children starting at a young age. This interaction has the potential to enhance literacy development. Additionally, educated mothers are inclined to offer a greater array of educational materials, including books, educational toys and learning exercises, which can inspire children’s interest in reading and mathematics early on. In general, mothers’ educational background significantly influences children’s literacy-numeracy development by shaping various aspects of the home environment, parenting practices, and educational opportunities.

The results from this study is based on observational data to infer causal relationship between maternal education and literacy-numeracy development. We fully acknowledge that, like all observational analyses, this study is potentially subject to hidden biases arising from unobserved confounders such as maternal cognitive ability, parenting style, or home learning environment. While we adjusted for a comprehensive set of socio-demographic covariates, unmeasured factors that influence both maternal education and child development may still exist. However, the sensitivity analysis confirmed that the estimated treatment effect was robust to potential hidden biases. Also, as with all observational approaches, the estimated effects should therefore be interpreted as strengthened causal evidence rather than definitive causal effects.

Since this study is based on cross-sectional data, it cannot firmly establish the temporal direction of the observed associations. Although maternal education likely predates the development of literacy-numeracy skills in 3–4-year-old children, longitudinal data would be needed to more robustly confirm the directional and persistence of these effects over time.

Finally, the literacy-numeracy outcome used in this study is based on the MICS Early Childhood Development Index and captures basic foundational skills such as letter recognition, word reading, and number identification. While this measure is internationally comparable, it represents a relatively crude indicator of early cognitive development and should be interpreted as reflecting early school readiness rather than complete literacy or numeracy competence.

## 5. Conclusions

Overall, the findings of the research indicate a strong causal connection between maternal education and the early childhood development of literacy-numeracy skills in Bangladeshi children aged 3–4 years. Considering that 71.46% of children in Bangladesh were not meeting developmental milestones in literacy-numeracy, it may be more effective to allocate efforts and resources towards improving maternal education. Government authorities and policymakers can take steps to increase the enrollment of female students in quality education. The enactment of policies specifically aimed at offering education to girls, who will eventually become mothers, can act as an effective and pragmatic intervention, contributing to Bangladesh’s progress toward achieving the SDG 4.2 target which emphasises universal access to quality early childhood development, care and pre-primary education.

In addition to investing in formal female education, targeted interventions could further enhance child development outcomes. For instance, community-based early childhood education centers can support children’s school readiness, particularly in under-served areas. Parenting support programs that coach mothers-especially those with limited education-on early literacy and numeracy stimulation could directly improve the home learning environment. Moreover, conditional cash transfer schemes that incentivise girls’ school attendance may help break inter-generational cycles of low education and poor child development. These complementary strategies may provide more immediate and context-sensitive avenues for improving early childhood outcomes in Bangladesh.
